# Clinical signs, management, and survival of 278 dogs diagnosed with insulinoma under primary veterinary care in the United Kingdom

**DOI:** 10.1093/jvimsj/aalag045

**Published:** 2026-03-13

**Authors:** Kasper Kraai, Dan G O’Neill, Lucy J Davison, Dave C Brodbelt, Sara Galac, Floryne O Buishand

**Affiliations:** Department of Clinical Sciences, Faculty of Veterinary Medicine, Utrecht University, Utrecht 3584 CM, The Netherlands; Pathobiology and Population Sciences, The Royal Veterinary College, Hatfield AL9 7TA, United Kingdom; Clinical Science and Services, The Royal Veterinary College, Hatfield AL9 7TA, United Kingdom; Department of Physiology, Anatomy and Genetics, University of Oxford, Oxford OX1 3PT, United Kingdom; Pathobiology and Population Sciences, The Royal Veterinary College, Hatfield AL9 7TA, United Kingdom; Department of Clinical Sciences, Faculty of Veterinary Medicine, Utrecht University, Utrecht 3584 CM, The Netherlands; Clinical Science and Services, The Royal Veterinary College, Hatfield AL9 7TA, United Kingdom

**Keywords:** canine, pancreatic neuroendocrine tumor, diazoxide, glucocorticoids, hyperinsulinemia, hypoglycemia, VetCompass

## Abstract

**Background:**

Insulinoma is the most commonly diagnosed endocrine tumor of the pancreas in dogs. Current literature has predominately focused on referral management of insulinoma in dogs.

**Hypothesis/Objectives:**

To describe clinical signs, management, and survival and to explore risk factors associated with clinical management undertaken for insulinoma in dogs under primary veterinary care in the United Kingdom.

**Animals:**

Two hundred seventy-eight insulinoma cases identified from 225 0741 VetCompass study dogs within the United Kingdom in 2019.

**Methods:**

Nested cohort study. Insulinoma cases were identified by manual review of electronic health records. Multivariable logistic regression was used to identify risk factors associated with clinical management. The Kaplan–Meier method with log rank test and multivariable Cox regression were used to identify risk factors associated with survival.

**Results:**

Epileptiform seizures, weakness, collapse/syncope, and muscle fasciculations were the most commonly reported clinical signs. Spaniel breed dogs (OR 2.43, 95% CI 1.02-5.79), dogs with epileptiform seizures (OR 2.15, 95% CI 1.15-4.02) and referred dogs (OR 4.85, 95% CI 2.42-9.72) had increased odds of undergoing surgery, compared to non-spaniel breed dogs, dogs without epileptiform seizures, and non-referred dogs. Compared to dogs treated solely medically, dogs treated surgically had a lower hazard (HR 0.49, 95% CI 0.32-0.77) of dying. Referred dogs had a longer median survival time (673 days, IQR 221-1139) than non-referred dogs (275 days, IQR 55-735) (*P <* .001).

**Conclusions and clinical importance:**

This study identified that referral and surgical treatment are associated with improved clinical outcomes for dogs with insulinoma presenting to primary veterinary care.

## Introduction

Insulinoma is a pancreatic beta-cell tumor that is the most common neuroendocrine tumor of the canine pancreas.^1^ The annual prevalence and incidence rate in UK primary care practices are 4 per 100 000 and 3 per 100 000 dogs, respectively.^2^ Reported breed predispositions include Dogue de Bordeaux, German Pointer, Flat-Coated Retriever, Boxer, and West Highland White Terrier.^2^ The most commonly reported clinical signs of hyperinsulinemia-induced hypoglycemia clinical signs include epileptiform seizures, weakness, and syncope.^1^^,^^3–5^ The median age at diagnosis is 8.5-10 years (range of 3-15 years).^2^^,^^5^^,^^6^ Diagnosis is based on concurrent hypoglycemia and inappropriately high insulin concentration in blood.^5^ Contrast-enhanced computed tomography (CT) is currently the most sensitive modality for localizing primary and metastatic insulinomas.^5^^,^^7–9^ Treatment options for insulinoma include medical management, surgical therapy, or a combination of the two. The reported median survival time (MST) of medically treated dogs varies between studies depending on the medication used but ranges between 67 and 399 days.^4^^,^^10–12^ Surgery offers a significantly longer MST compared to medical treatment, with reported MST of dogs treated surgically varying from 180 to 762 days.^10^^,^^13–15^ Until now, studies describing clinical management of insulinoma in dogs, predominantly focused on cases from referral centers, whereas data from primary veterinary care practices remain largely absent. Insulinoma in dogs is likely under-recognized in the primary care setting because clinical signs resulting from hypoglycemia can mimic neurological, cardiovascular, and other metabolic diseases.^5^^,^^6^ Establishing evidence-based insights from primary care populations is therefore crucial to understanding real-world insulinoma diagnostic pathways and management outcomes.

Prognostic factors previously associated with insulinoma in dogs include age, Tumor Node Metastasis stage, tumor size, Ki67 index, and post-operative blood glucose concentration, but their significance has varied across studies.^13^^,^^14^^,^^16^^,^^17^ Hence, further research into risk factors for treatment selection and survival of dogs diagnosed with insulinoma in primary care is needed. Identifying determinants that influence whether dogs are referred, undergo surgery or receive medical treatment could improve prognostication and case management in primary care practice.

This study aimed to report the clinical signs, diagnostic investigations, and clinical management of dogs with insulinoma under primary veterinary care in the United Kingdom. Specific objectives were to identify risk factors for clinical management decision-making for dogs diagnosed with insulinoma (surgical versus non-surgical treatment and referral versus non-referral) and to investigate survival after diagnosis of insulinoma in dogs presented to primary care practices in the United Kingdom. Together, these findings aim to enhance the evidence base available to primary care veterinarians, supporting informed decision-making for owners of dogs diagnosed with insulinoma.

## Materials and methods

This study was an extension of an earlier VetCompass study of insulinoma in dogs.^2^ In summary, insulinoma cases were defined as having at least one of the following inclusion criteria:

1. Evidence of a final clinical diagnosis of insulinoma recorded in available electronic health records (EHRs).
2. Histopathological confirmation of insulinoma reported in available EHRs.
3. Concurrent occurrence of blood glucose < 4.2 mmol/L, plasma insulin > 10 μU/mL and matching clinical signs of hypoglycemia in available EHRs.
4. Evidence of concurrent hypoglycemia, a pancreatic mass lesion on diagnostic imaging and matching clinical signs of hypoglycemia in available EHRs.

A total of 278 dogs diagnosed with insulinoma under primary UK veterinary care in 2019 were included, with follow-up to August 31, 2023. Detailed case-finding procedures and population characteristics have previously been published.^2^^,^^18^ A nested cohort design within the previous study was used for the current survival analysis as well as to report on clinical signs, diagnostic tests used for insulinoma diagnosis and evaluation of metastatic disease and treatment(s).

The following variables were collected as described in the published study^2^: Sex, Neuter, Breed, Terrier (distinguishing terrier breeds assigned to the breed group “terrier” after a combined classification according to the Kennel Club and VeNom Coding Group and “non-terrier” breeds)*,* Median adult bodyweight*,* Purebred*,* and Median adult bodyweight in relation to the median for the sex/breed.^2^ Additional clinical variables, including age at time of diagnosis, clinical signs, diagnostic tests for insulinoma or metastatic evaluation, treatment modality, referral status, and survival, were extracted and classified as described in the Supplementary Appendix.

Continuous variables that were normally distributed were summarized using mean (SD) and non-normally distributed data were reported as median (IQR and range). CIs were derived from standard errors, based on approximation to the binomial distribution. Risk factor analysis used binary logistic regression modeling. Risk factors with liberal association (*P*-value <.2) in univariable modeling were taken forwards for consideration in multivariable modeling. Using manual backward elimination, a final multivariable binary logistic regression model was composed including variables with a *P-*value of <.05. Variables derived from the breed variable were excluded from an initial breed-focused model and instead individually replaced the *Breed* variable in the final breed-focused multivariable model to evaluate their effects after taking account of the other variables.^19^ The Kaplan–Meier method with log rank test and multivariable Cox regression were used to identify risk factors associated with survival with *P*-value <.05 considered significant. Detailed descriptions of the statistical analyses are provided in the Supplementary Appendix.

## Results

### Descriptive results

The study included 278 dogs diagnosed with insulinoma under primary veterinary care in the UK. Of the 278 insulinoma cases, 123 (44.2%) were male (52.0% neutered) and 153 (55.0%) were female (73.2% neutered). Age at time of diagnosis was available for 268 of 278 (96.4%) dogs. The median age at time of diagnosis was 10.4 years (IQR 8.7-12.2, range 3.2-16.2). Median adult bodyweight was available for 252 of 278 (90.7%) dogs. The median of the median adult bodyweight was 18.9 kg (IQR 9.3-28.4, range 3.4-68.3). The most common breeds among the cases were crossbreed (*n* = 67, 24.1%), West Highland White Terrier (*n* = 31, 11.1%), and Jack Russell Terrier (*n* = 24, 8.6%).^2^

### Clinical signs and diagnostic tests

**Figure 1:**
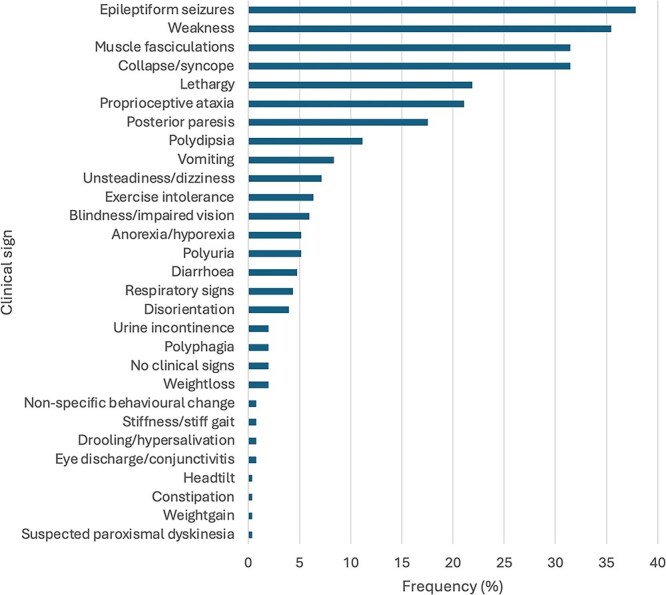
Presenting clinical signs at the time of diagnosis in dogs diagnosed with insulinoma in primary care practices in the United Kingdom in 2019 within VetCompass (*n* = 251).

At least one clinical sign at presentation was recorded for 251 out of 278 dogs (90.3%). Of these, the most commonly recorded clinical signs were epileptiform seizures (95/251, 37.8%), weakness (89/251, 35.5%), collapse/syncope (79/251, 31.5%), and muscle fasciculations (79/251, 31.5%) (Figure 1). Of the dogs with at least one recorded clinical sign at presentation, 105 out of 251 (41.8%) exhibited only 1 out of 4 most common signs, 87 out of 251 (34.7%) showed 2, and 21 out of 251 (8.4%) displayed 3 of these clinical signs. No dogs had all 4 most common clinical signs, while 38 out of 251 (15.1%) showed none of these 4 common signs, but did have another clinical sign recorded. Of the dogs that exhibited a combination of 2 or more of the 4 most common signs, the most common combinations of signs were weakness and collapse/syncope in 21 out of 108 (19.4%), weakness and muscle fasciculations in 18 out of 108 (16.7%), and weakness and epileptiform seizures in 15 out of 108 (13.9%) dogs (Table S1).

At least one diagnostic test was recorded under primary care for 205 out of 278 (73.7%) cases. Of these, concurrent blood glucose and serum insulin measurements were recorded for 190 out of 205 (92.7%) cases. Ultrasonography was used in 72 out of 205 cases (35.1%) to evaluate presence or absence of a pancreatic mass, and CT was used in 18 out of 205 cases (8.8%). In 10 out of 205 cases (4.9%), exploratory laparotomy or laparoscopy was performed as a diagnostic modality, with insulinoma confirmed by histopathology in 5 out of 10 (50%) of these surgical investigations. In 3 out of 205 cases (1.5%), cytologic interpretation of ultrasound-guided fine needle aspirations of pancreatic nodules was used. No diagnostic tests were recorded under primary care for 73 out of 278 (26.3%) dogs. Of these, 49 out of 73 (67.1%) dogs were referred before any diagnostic tests were performed, 17 out of 73 (23.3%) dogs had been previously diagnosed under the care of another practice, and the reason for the absence of diagnostic testing was unclear from the EHRs of the remaining 7 out of 73 (9.6%) dogs. The position of the insulinoma within the pancreas was recorded in 36 out of 278 (12.9%) cases. Of these cases, the insulinoma was located in the left limb for 14 (38.9%) dogs, in the right limb for 18 (50%) dogs and in the pancreatic body for four (11.1%) dogs. Presence of potential metastatic lesions was evaluated using diagnostic imaging in 71 out of 278 (25.5%) cases. Ultrasonography was used in 61 out of 71 (85.9%) cases, CT was used in 18 out of 71 (25.4%) cases, radiography was used in 8 out of 71 (11.3%) cases. The outcomes of these tests were not recorded in 10 out of 71 (14.1%) cases. No metastatic lesions were identified in 41 out of 71 (57.7%) dogs and suspected metastatic lesions were detected in the abdominal lymph nodes in 14 out of 71 (19.7%) dogs, in the liver in 11 out of 71 (15.5%) dogs, in the spleen in 3 out of 71 (4.2%) dogs, in the duodenum in 1 out of 71 (1.4%) dog, in the mediastinum in 1 out of 71 (1.4%) dog, and in the left thoracic cavity cranial to the heart (tissue unspecified) in 1 out of 71 (1.4%) dog.

### Referral care and management

From 263 out of 278 (94.6%) dogs with information recorded on whether the dog was referred, 134 out of 263 dogs (51.0%) received referral care. Of the referred dogs, 90 out of 134 (67.2%) were referred for both diagnostics and treatment, 14 out of 134 (10.4%) dogs were referred for diagnostics only, 5 out of 134 (3.7%) dogs were referred for treatment only, and 25 out of 134 (18.7%) dogs were not physically referred, but specialist advice was obtained.

Information regarding treatment was recorded for 271 out of 278 dogs (97.5%). After diagnosis, 25 out of 271 (9.2%) dogs were euthanized without any treatment and 14 out of 271 (5.2%) dogs were conservatively managed with restricted exercise and dietary changes but without any active medical or surgical treatment. The remaining 232 out of 271 (85.6%) dogs received either medical treatment, surgical treatment, or a combination of both: 20 out of 271 (7.4%) dogs had surgery only, 49 out of 271 (18.1%) dogs had both surgery and received medical treatment and 163 out of 271 (65.3%) dogs received medical treatment only. Of the surgically treated dogs, 19 out of 69 (27.5%) dogs had one surgery at primary care practice, 41 out of 69 (59.4%) dogs had one surgery at a referral center and the location of surgery was unrecorded for 4 out of 69 (5.8%) dogs. Five dogs out of 69 dogs (7.2%) underwent surgery twice, of which 1 dog had initial surgery under primary care and again later under referral care and 1 dog had both surgeries under primary care. Within the 49 cases that received both surgical and medical treatment, different orders of treatment modalities were used: 9 out of 49 (18.4%) dogs first received medical treatment followed by surgery, whereas 40 out of 49 (81.6%) dogs first had surgery and received medical treatment afterwards. Additionally, 3 out of 49 (6.1%) dogs first received medical treatment, followed by surgery and continuation of medical management after surgery and 1 out of 49 (2.0%) dogs had surgery, received medical treatment, had surgery again, and received medical treatment afterwards.

Of the 212 dogs that received medical treatment (with or without surgery), 210 out of 212 (99.1%) received glucocorticoids, 18 out of 212 (8.5%) received toceranib phosphate, 15 out of 212 (7.1%) received diazoxide, and 2 out of 212 (0.9%) received somatostatin analogues. No dogs received glucagon as treatment for hypoglycemia or streptozotocin as treatment for insulinoma. Of all medically managed dogs, 205 out of 212 (96.7%) dogs started medical treatment with single drug therapy. In 6 dogs (2.8%), treatment was initiated with a combination of glucocorticoids and toceranib phosphate and one dog (0.5%) was initially treated with both glucocorticoids and diazoxide.

### Risk factor analysis for treatment modality

Univariable logistic regression modeling identified six variables liberally associated with treatment modality (*P* < .20): neuter, terrier breed, spaniel breed, age at time of diagnosis, referral status, and epileptiform seizures (Table 1). Manual backward elimination of the multivariable binary logistic regression model resulted in spaniel breed, age at time of diagnosis, referral status, and epileptiform seizures as final variables in the model being significantly associated (*P* < .05) with treatment modality (Table 2). Spaniel breed dogs had greater odds (OR 2.43, 95% CI, 1.02-5.79) of receiving surgical treatment compared to non-spaniel breed dogs. Referred dogs had greater odds (OR 4.85, 95% CI, 2.42-9.72) for undergoing surgery compared to non-referred dogs and dogs with epileptiform seizures also had higher odds (2.15 95% CI, 1.15-4.02) for having surgery compared to dogs without epileptiform seizures. In contrast, dogs aged ≥9 years at time of diagnosis had lower odds (OR 0.45 95% CI, 0.24-0.87) for receiving surgical treatment compared to dogs < 9 years. The final multivariable model showed no evidence of poor fit (Hosmer-Lemeshow *P* = .676). The area under the Receiver Operating Characteristic curve of the final model was 0.765, indicating good discrimination.

**Table 1:** Univariable logistic regression results for risk factors evaluated for treatment outcome (surgical versus non-surgical management) of 278 dogs diagnosed with insulinoma attending UK primary care veterinary practices in 2019 within VetCompass.

Variable	Number of dogs untreated or non-surgically treated (%)	Number of dogs surgically treated (%)	Odds ratio	95% CI	*P*-value
**Sex**					.478
**Female**	112 (53.6)	41 (59.4)	Baseline		
**Male**	96 (45.9)	27 (39.1)	0.77	0.44-1.34	.354
**Unrecorded**	1 (0.5)	1 (1.5)	2.73	0.17-44.69	.481
**Neuter**					**.067**
**Entire**	83 (39.7)	17 (24.6)	Baseline		
**Neutered**	125 (59.8)	51 (73.9)	1.99	1.08-3.69	**.028**
**Unrecorded**	1 (0.5)	1 (1.5)	4.88	0.29-81.95	.271
**Median adult bodyweight**					.545
**<10 kg**	57 (27.3)	17 (24.6)	Baseline		
**10-20 kg**	41 (19.6)	16 (23.2)	1.31	0.59-2.89	.506
**20-30 kg**	51 (24.4)	21 (30.4)	1.38	0.66-2.90	.395
**>30 kg**	41 (19.6)	8 (11.6)	0.65	0.26-1.66	.372
**Unrecorded**	19 (9.1)	7 (10.1)	1.24	0.45-3.43	.685
**Breed**					.371
**Crossbreed**	53 (25.4)	14 (20.3)	Baseline		
**West Highland White Terrier**	25 (12.0)	6 (8.7)	0.91	0.31-2.64	.860
**Jack Russell Terrier**	19 (9.1)	5 (7.2)	1.00	0.32-3.32	.995
**Boxer**	16 (7.7)	5 (7.2)	1.18	0.37-3.79	.777
**English Springer Spaniel**	9 (4.3)	8 (11.6)	3.37	1.10-10.31	**.034**
**Border Collie**	6 (2.9)	4 (5.8)	2.52	0.63	.194
**Staffordshire Bull Terrier**	9 (4.3)	0	0	0	.999
**German Pointer**	6 (2.9)	1 (1.4)	0.63	0.07-5.68	.681
**Labrador Retriever**	5 (2.4)	1 (1.4)	0.76	0.08-7.02	.807
**English Cocker Spaniel**	2 (1.0)	1 (1.4)	7.57	1.26-45.65	**.027**
**Golden Retriever**	3 (1.4)	2 (2.9)	2.52	0.38-16.60	.335
**Other breeds**	56 (26.8)	22 (31.9)	1.28	0.59-2.82	.532
**Purebred**					.670
**Crossbreed**	53 (25.4)	14 (20.3)	Baseline		
**Designer**	7 (3.3)	2 (2.9)	1.08	0.20-5.79	.927
**Purebred**	149 (71.3)	53 (76.8)	1.35	0.69-2.62	.382
**Median adult bodyweight in relation to median for the sex/breed**					.488
**At or below median for the sex/breed**	74 (35.4)	19 (27.5)	Baseline		
**Above median for the sex/breed**	116 (55.5)	43 (62.3)	1.44	0.78-2.67	.241
**Missing data**	19 (9.1)	7 (10.1)	1.44	0.53-3.91	.480
**Terrier**					.180
**Non-terrier**	146 (69.9)	54 (78.3)	Baseline		
**Terrier**	63 (30.1)	15 (21.7)	0.64	0.34-1.23	.180
**Spaniel**					**.001**
**Non-spaniel**	194 (92.8)	54 (78.3)	Baseline		
**Spaniel**	15 (7.2)	15 (21.7)	3.59	1.65-7.81	**.001**
**Age at time of diagnosis**					**.002**
**<9**	45 (21.5)	30 (43.5)	Baseline		
**≥9**	157 (75.1)	36 (52.2)	0.34	0.19-0.62	**<.001**
**Unrecorded**	7 (3.3)	3 (4.3)	0.64	0.15-2.68	.545
**Referral status**					**<.001**
**Not referred**	116 (55.5)	13 (18.8)	Baseline		
**Referred**	82 (39.2)	52 (75.4)	5.66	2.90-11.06	**<.001**
**Missing data**	11 (5.3)	4 (5.8)	3.25	0.90-11.67	.071
**Epileptiform seizures**					
**Absent**	146 (69.9)	37 (53.6)	Baseline		**.015**
**Present**	63 (30.1)	32 (46.4)	2.00	1.15-3.50	**.015**
**Weakness**					
**Absent**	140 (67.0)	49 (71.0)	Baseline		.534
**Present**	69 (33.0)	20 (29.0)	0.83	0.4601.50	.534
**Collapse/syncope**					.621
**Absent**	148 (70.8)	51 (73.9)	Baseline		
**Present**	61 (29.2)	18 (26.1)	0.86	0.46-1.58	.621
**Muscle fasciculations**					.268
**Absent**	146 (69.9)	53 (76.8)	Baseline		
**Present**	63 (30.1)	16 (23.2)	0.70	0.37-1.32	.268
**Total**	209 (100.0)	69 (100.0)			

Significant *P*-values (*P* < .05) are in bold.

**Table 2:** Multivariable logistic regression results for risk factors evaluated for treatment outcome (surgical versus non-surgical management) of 278 dogs diagnosed with insulinoma attending UK primary care veterinary practices in 2019 within VetCompass.

Variable	Odds ratio	95% CI	*P*-value
**Spaniel**			**.044**
**Non-spaniel**	Baseline		
**Spaniel**	2.43	1.02-5.79	**.044**
**Age at time of diagnosis**			**.049**
**<9**	Baseline		
**≥9**	0.45	0.24-0.87	**.017**
**Unrecorded**	0.93	0.06-12.54	.959
**Referral status**			**<.001**
**Not referred**	Baseline		
**Referred**	4.85	2.42-9.72	**<.001**
**Missing data**	3.38	0.34-33.77	.300
**Epileptiform seizures**			**.016**
**Absent**	Baseline		
**Present**	2.15	1.15-4.02	**.016**

Significant *P*-values (*P* < .05) are in bold.

### Risk factor analysis for referral status

Univariable logistic regression identified five variables liberally associated with referral *(P* < .20): breed, terrier breed, spaniel breed, age at time of diagnosis, and weakness (Table 3). Manual backward elimination of the multivariable binary logistic regression model resulted in age at time of diagnosis and spaniel breed as final variables being significantly (*P <* .05) associated with referral (Table 4). Dogs aged ≥9 years at time of diagnosis had lower odds (OR 0.45, 95% CI, 0.26-0.79) of being referred compared with dogs < 9 years. In contrast, spaniel breed dogs had higher odds (OR 2.73, 95% CI, 1.15-6.49) of being referred compared to non-spaniel breed dogs. The final multivariable model showed no evidence of poor fit (Hosmer-Lemeshow *P =* .983). The area under the Receiver Operating Characteristic curve of the final model was 0.636, indicating satisfactory discrimination.

**Table 3:** Univariable logistic regression results for risk factors associated with being referred among 278 dogs diagnosed with insulinoma attending UK primary care veterinary practices in 2019 within VetCompass.

Variable	Number of non-referred dogs (%)	Number of referred dogs (%)	Odds ratio	95% CI	*P*-value
**Sex**					.858
**Female**	77 (53.5)	76 (56.7)	Baseline		
**Male**	66 (45.8)	57 (42.5)	0.88	0.54-1.41	.582
**Unrecorded**	1 (0.7)	1 (0.7)	1.01	0.06-16.49	.993
**Neuter**					.430
**Entire**	57 (39.6)	43 (32.1)	Baseline		
**Neutered**	86 (59.7)	90 (67.2)	1.39	0.85-2.27	.194
**Unrecorded**	1 (0.7)	1 (0.7)	1.33	0.08-21.80	.844
**Median adult bodyweight**					.733
**<10 kg**	42 (29.2)	32 (23.9)	Baseline		
**10-20 kg**	29 (20.1)	28 (20.9)	1.27	0.63-2.54	.503
**20-30 kg**	33 (22.9)	39 (29.1)	1.55	0.81-2.98	.188
**>30 kg**	27 (18.8)	22 (16.4)	1.07	0.52-2.21	.856
**Unrecorded**	13 (9.0)	13 (9.7)	1.31	0.54-3.22	.552
**Breed**					.199
**Crossbreed**	38 (26.4)	29 (21.6)	Baseline		
**West Highland White Terrier**	21 (14.6)	10 (7.5)	0.62	0.26-1.53	.302
**Jack Russell Terrier**	12 (8.3)	12 (9.0)	1.3	0.52-3.34	.571
**Boxer**	13 (9.0)	8 (6.0)	0.81	0.30-2.20	.675
**English Springer Spaniel**	4 (2.8)	13 (9.7)	4.26	1.26-14.43	**.020**
**Border Collie**	6 (4.2)	4 (3.0)	0.87	0.23-3.38	.845
**Staffordshire Bull Terrier**	6 (4.2)	3 (2.2)	0.66	0.15-2.84	.572
**German Pointer**	4 (2.8)	3 (2.2)	0.98	0.20-4.74	.983
**Labrador Retriever**	2 (1.4)	4 (3.0)	2.62	0.50-15.31	.285
**English Cocker Spaniel**	1 (0.7)	5 (3.7)	6.55	0.73-59.17	.094
**Golden Retriever**	2 (1.4)	3 (2.2)	1.97	0.31-12.54	.475
**Other breeds**	35 (24.3)	40 (29.9)	1.50	0.77-2.91	.232
**Purebred**					.619
**Crossbreed**	38 (26.4)	29 (21.6)	Baseline		
**Designer**	5 (3.5)	4 (3.0)	1.05	0.26-4.25	.947
**Purebred**	101 (70.1)	101 (75.4)	1.31	0.75-2.29	.341
**Median adult bodyweight in relation to median for the sex/breed**					.623
**At or below mean adult BW**	52 (36.1)	41 (30.6)	Baseline		
**Above mean adult BW**	79 (54.9)	80 (59.7)	1.28	0.77-2.15	.340
**Missing data**	13 (9.0)	13 (9.7)	1.27	0.53-3.03	.593
**Terrier**					.136
**Non-terrier**	98 (68.1)	102 (76.1)	Baseline		
**Terrier**	46 (31.9)	32 (23.9)	0.67	0.39-1.14	.136
**Spaniel**					**.005**
**Non-spaniel**	136 (94.4)	112 (83.6)	Baseline		
**Spaniel**	8 (5.6)	22 (16.4)	3.34	1.43-7.79	**.005**
**Age at time of diagnosis**					**.008**
**<9**	26 (18.1)	49 (36.6)	Baseline		
**≥9**	108 (75.0)	85 (63.4)	0.42	0.24-0.73	**.002**
**Unrecorded**	10 (6.9)	0	0	0	.999
**Epileptiform seizures**					.417
**Absent**	98 (68.1)	85 (63.4)	Baseline		
**Present**	46 (31.9)	49 (36.6)	1.23	0.75-2.02	.417
**Weakness**					.069
**Absent**	105 (72.9)	84 (62.7)	Baseline		
**Present**	39 (27.1)	50 (37.3)	1.60	0.97-2.66	.069
**Collapse/syncope**					.297
**Absent**	107 (74.3)	92 (68.7)	Baseline		
**Present**	37 (25.7)	42 (31.3)	1.32	0.78-2.23	.297
**Muscle fasciculations**					.774
**Absent**	102 (70.8)	97 (72.4)	Baseline		
**Present**	42 (29.2)	37 (27.6)	0.93	0.55-1.56	.774
**Total**	144 (100.0)	134 (100.0)			

Significant *P*-values (*P* < .05) are in bold.

**Table 4:** Multivariable logistic regression results for risk factors associated with being referred among 278 dogs diagnosed with insulinoma attending UK primary care veterinary practices in 2019 within VetCompass.

Variable	Odds ratio	95% CI	*P*-value
**Spaniel**			**.023**
**Non-spaniel**	Baseline		
**Spaniel**	2.72	1.15-6.44	**.023**
**Age at time of diagnosis**			**.021**
**<9**	Baseline		
**≥9**	0.45	0.26-0.79	**.006**
**Unrecorded**	0	0	.999

Significant *P*-values (*P* < .05) are in bold.

### Survival

Ten out of 278 cases (3.6%) had no available date of first insulinoma diagnosis and were excluded from survival analysis. The MST of the 268 included insulinoma cases was 291 days (IQR 72-712 days, range 0-2299 days) (Figure S1). Overall, 209 out of 278 (75.2%) dogs had a date of death recorded in the available EHR, with the remaining cases either still alive at the end of the study or lost to follow up. The cases without a date of death consisted of 34 out of 278 (12.2%) dogs that were still alive at the end of the study period and 35 out of 278 (12.6%) dogs that were lost to follow up. Within the group of dogs with a date of death recorded, 189 out of 209 (90.4%) dogs were euthanized, 15 out of 209 (7.2%) died unassisted, and for 5 (2.4%) dogs the nature of the death was unclear in the EHRs.

The death was related to insulinoma in 151 out of 209 (72.2%) dogs, not related to insulinoma in 16 out of 209 (7.7%) dogs, and for 42 out of 209 (20.1%) dogs, it was unclear in the EHRs whether the death was related to insulinoma or not. Six out of 151 (4.0%) dogs had no available date of first insulinoma diagnosis and were excluded for survival analysis for this group. MST of the cases with a death related to insulinoma (*n* = 145) was 168 days (IQR 46-462 days, range 0-2205 days).

**Figure 2:**
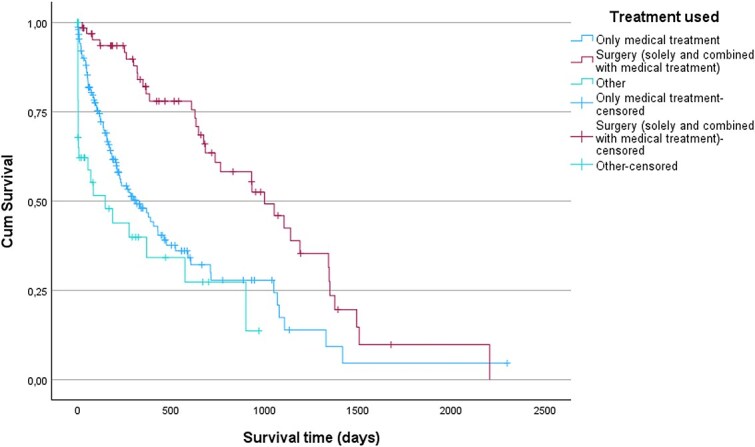
Kaplan–Meier curves for dogs diagnosed with insulinoma without treatment, or treated with medical therapy only, or with surgery. Vertical dashes indicate censoring of dogs. There was a significant difference in survival time (*P* < .001) between surgically treated cases and cases that were only medically managed or did not receive treatment.

**Figure 3:**
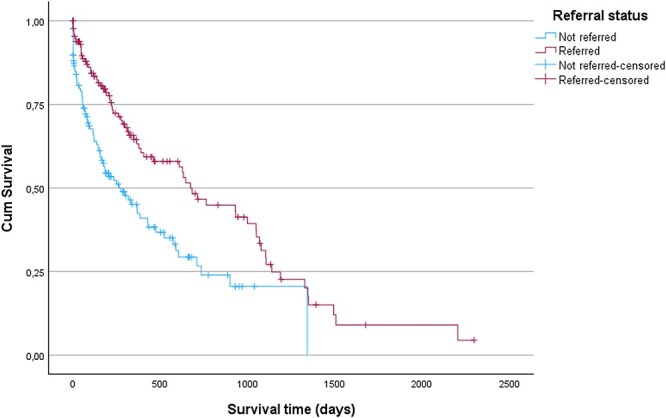
Kaplan–Meier curves for dogs diagnosed with insulinoma that were either referred or not referred. Vertical dashes indicate censoring of dogs. There was a significant difference in survival time (*P* < .001) between cases that were referred and cases that were not referred.

**Figure 4:**
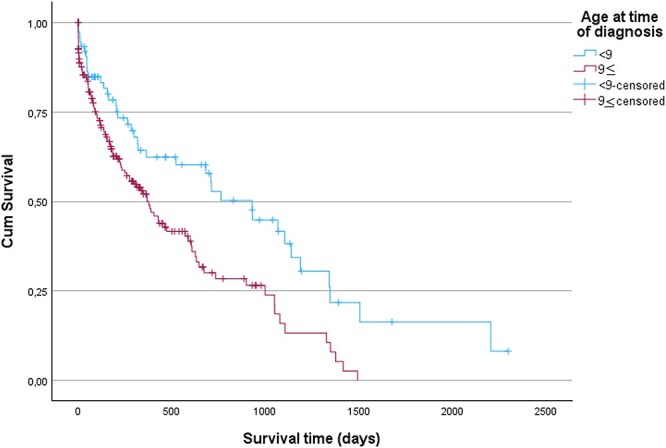
Kaplan–Meier curves for dogs diagnosed with insulinoma aged < 9 or ≥9 years at time of diagnosis. Vertical dashes indicate censoring of dogs. There was a significant difference in survival time (*P =* .001) between cases aged < 9 or ≥ 9 years at time of diagnosis.

MST for surgically treated cases (either being solely surgically treated or in combination with medical treatment) was 999 days (IQR 627-1349 days, range 24-2205 days, *n =* 34) and significantly longer than for cases that were only medically managed (311 days, IQR 114-1049 days, range 1-1417 days, *n* = 86), or cases that received no treatment (147 days, IQR 1-899 days, range 0-899 days, *n* = 25) (*P* = < .001) (Figure 2). MST for referred cases was 673 days (IQR 221-1139 days, range 0-2205 days, *n =* 68) and significantly longer than MST for cases not referred (275 days, IQR 55-735 days, range 0-1342 days, *n* = 129) (*P* < .001) (Figure 3). MST for dogs < 9 years of age at time of diagnosis was 930 days (IQR 211-1346 days, range 0-2205 days, *n =* 39) and significantly longer than MST for dogs aged ≥9 years at time of diagnosis (377 days, IQR 96-999 days, range 0-1493 days, *n =* 106) (*P* = .001) (Figure 4).

Univariable Cox regression modeling identified 6 variables liberally associated with survival (*P* < .20): neuter, spaniel breed, age at time of diagnosis, referral status, treatment used, and weakness (Table 5). Manual backward elimination of the multivariable Cox regression model resulted in age at time of diagnosis, treatment modality, and referral status as final variables in the model. Age at time of diagnosis, treatment modality and referral status were significantly associated (*P* < .05) with survival (Table 6). Dogs aged ≥9 years at time of diagnosis had a higher hazard (HR 1.70, 95% CI, 1.14-2.54) of dying compared to dogs aged < 9 years. Dogs treated surgically (either being solely surgically treated or in combination with medical treatment) had a lower hazard (HR 0.49, 95% CI, 0.32-0.77) of dying compared to medically-only treated dogs, whereas dogs that did not receive treatment had an increased hazard (HR 1.71, 95% CI, 1.08-2.71) of dying. Dogs with missing data regarding referral had a lower hazard (HR 0.19, 95% CI, 0.04-0.81) of dying compared to non-referred dogs. The likelihood-ratio test indicated good model fit (*χ^2^*(5) = 46.364, *P <* .001).

**Table 5:** Univariable Cox proportional hazards regression results for risk factors associated with death related to insulinoma (*n =* 145) among 268 dogs diagnosed with insulinoma attending UK primary care veterinary practices in 2019 within VetCompass.

Variable	Number of dogs (%)	Hazard ratio	95% CI	*P*-value
**Sex**				.307
**Female**	74 (51.0)	Baseline		
**Male**	70 (48.3)	1.17	0.84-1.62	.349
**Unrecorded**	1 (0.7)	0.32	0.04-2.29	.254
**Neuter**				.166
**Entire**	50 (34.5)	Baseline		
**Neutered**	94 (64.8)	0.77	0.54-1.10	.146
**Unrecorded**	1 (0.7)	0.24	0.03-1.77	.161
**Median adult bodyweight**				.974
**<10 kg**	36 (24.8)	Baseline		
**10-20 kg**	33 (22.8)	1.01	0.63-1.62	.982
**20-30 kg**	40 (27.6)	1.07	0.68-1.68	.764
**>30 kg**	26 (17.9)	1.15	0.70-1.91	.579
**Unrecorded**	10 (6.9)	1.17	0.58-2.36	.664
**Breed**				.721
**Crossbreed**	31 (21.4)	Baseline		
**West Highland White terrier**	16 (11.3)	1.34	0.73-2.46	.339
**Jack Russell terrier**	12 (8.3)	0.98	0.50-1.93	.958
**Boxer**	12 (8.3)	1.15	0.59-2.26	.679
**English Springer Spaniel**	12 (8.3)	0.81	0.41-1.60	.546
**Border Collie**	6 (4.1)	1.32	0.55-3.16	.539
**Staffordshire Bull Terrier**	5 (3.4)	2.57	0.99-6.65	.052
**German Pointer**	3 (2.1)	1.11	0.34-3.65	.860
**Labrador Retriever**	2 (1.4)	1.55	0.37-6.54	.549
**English Cocker Spaniel**	3 (2.1)	0.58	0.18-1.92	.372
**Golden Retriever**	4 (2.8)	1.20	0.42-3.44	.733
**Other breeds**	39 (26.9)	1.30	0.81-2.09	.274
**Purebred**				.511
**Crossbreed**	31 (21.4)	Baseline		
**Designer**	6 (4.1)	1.64	0.68-3.94	.268
**Purebred**	108 (74.5)	1.16	0.77-1.73	.481
**Median adult bodyweight in relation to the median for the sex/breed**				.860
**At or below mean adult BW**	49 (33.8)	Baseline		
**Above mean adult BW**	86 (59.3)	0.92	0.65-1.31	.658
**Missing data**	10 (6.9)	1.06	0.54-2.09	.871
**Terrier**				.727
**Non-terrier**	109 (75.2)	Baseline		
**Terrier**	46 (24.8)	0.94	0.64-1.37	.727
**Spaniel**				.055
**Non-spaniel**	128 (88.3)	Baseline		
**Spaniel**	17 (11.7)	0.60	0.36-1.01	.055
**Age at time of diagnosis**				**.001**
**<9**	39 (26.9)	Baseline		
**≥9**	106 (73.1)	1.88	1.28-2.76	**.001**
**Unrecorded**	Excluded			
**Referral status**				**<.001**
**Not referred**	75 (51.7)	Baseline		
**Referred**	68 (46.9)	0.50	0.36-0.72	**<.001**
**Missing data**	2 (1.4)	0.22	0.05-0.91	**.037**
**Treatment used**				**<.001**
**Medical treatment**	86 (59.3)	Baseline		
**Surgery (solely or combined with medical treatment)**	34 (23.4)	0.42	0.28-0.64	**<.001**
**Other**	25 (17.2)	1.87	1.19-2.93	**.007**
**Location where surgery was performed^a^**	*n =* 36			.978
**Primary care practice**	11 (30.6)	Baseline		
**Referral center**	22 (61.1)	0.89	0.41-1.92	.769
**Both primary care practice and referral center**	1 (2.8)	1.03	0.13-8.21	.977
**Unrecorded**	2 (5.6)	0.67	0.09-5.31	.705
**Epileptiform seizures**				.115
**Absent**	97 (66.9)	Baseline		
**Present**	48 (33.1)	1.32	0.94-1.86	.115
**Weakness**				.695
**Absent**	93 (64.1)	Baseline		
**Present**	52 (35.9)	1.07	0.76-1.52	.695
**Collapse/syncope**				.985
**Absent**	100 (69.0)	Baseline		
**Present**	45 (31.0)	1.00	0.70-1.43	.985
**Muscle fasciculations**				.267
**Absent**	106 (73.1)	Baseline		
**Present**	39 (26.9)	1.24	0.85-1.79	.267

Significant *P*-values (*P* < .05) are in bold.

a Univariable Cox proportional hazards regression included 36/69 (52.2%) dogs that underwent surgery, 16/69 (23.2%) dogs were excluded because their life status was unclear, 10/69 (14.5%) dogs were excluded because they were still alive, 7/69 (10.1%) dogs were excluded because their death was unrelated to insulinoma.

**Table 6:** Final multivariable Cox proportional hazards regression results for risk factors associated with death related to insulinoma (*n =* 145) among 268 dogs diagnosed with insulinoma attending UK primary care veterinary practices in 2019 within VetCompass.

Variable	Hazard ratio	95% CI	*P*-value
**Age at time of diagnosis**			**.010**
**<9**	Baseline		
**≥9**	1.70	1.14-2.54	**.010**
**Referral status**			**.029**
**Not referred**	Baseline		
**Referred**	0.70	0.48-1.01	.054
**Missing data**	0.19	0.04-0.81	**.025**
**Treatment used**			
**Medical treatment**	Baseline		
**Surgery**	0.49	0.32-0.76	**.001**
**Other treatment**	1.71	1.08-2.70	**.022**

Significant *P*-values (*P* < .05) are in bold.

## Discussion

This study reports on clinical signs, management, survival, and risk factors associated with clinical management of insulinoma in dogs under primary veterinary care. The most common clinical signs identified were epileptiform seizures, weakness, collapse/syncope, and muscle fasciculations. Spaniel breed dogs, dogs with epileptiform seizures, and referred dogs had higher odds for undergoing surgery. Age at time of diagnosis and being a spaniel breed were associated with the odds of being referred. Surgically treated dogs had a significant longer MST than solely medically treated dogs and referred dogs had a significant longer MST than non-referred dogs.

The clinical signs described in the insulinoma cases in the current study were largely consistent with clinical signs described in previous studies, although the relative frequencies differed. In our primary care population, epileptiform seizures (37.8%), and weakness (35.5%) occurred less frequently compared to previous referral-based study populations, where these signs have been reported in 34%-69% and 29%-59.5% of cases, respectively.^4–6^^,^^13^^,^^14^^,^^20–22^ The percentage of cases with epileptiform seizures might have been underestimated in the current study because some cases with focal epileptiform seizures as a true clinical sign might have been recorded in the clinical notes as muscle fasciculations.^4^^,^^22^ Polyphagia was also reported less commonly in our study at 2%, compared with previous studies where it was reported in up to 9%.^4–6^^,^^13^^,^^14^^,^^20–22^ A possible explanation for these differences might be that previous studies predominantly focused on referral populations, where dogs with more severe or frequent clinical signs were likely overrepresented. In contrast, dogs with milder or intermittent signs, such as weight loss or subtle behavioral changes, might have remained within primary care. However, in our study, the likelihood of referral was not significantly associated with the presence of the most common clinical signs. It is also possible that more subtle clinical signs, such as polyphagia, were less readily recognized by owners or were not specifically investigated by primary care veterinarians compared with those in referral settings. In contrast, our primary care study did report several clinical signs that were not described in previous studies, such as respiratory signs, hypersalivation/drooling, eye discharge/conjunctivitis, stiffness, constipation, and head tilt.^4–6^^,^^13^^,^^14^^,^^20–22^ A possible explanation for the absence of these findings in earlier studies might be the relative rarity of these signs and therefore the larger scale of the current study required to identify them. Alternatively, these novel clinical observations might represent incidental or unrelated findings rather than true clinical features of insulinoma.

In our study, less than half of the cases had undergone imaging as part of the diagnostic work-up for insulinoma and only 25.5% had undergone imaging specifically performed to evaluate for the presence of metastatic disease. Incomplete or absent ultrasonography and CT reports resulted in missing data regarding the localization of pancreatic masses and the presence of metastases. When reported, the primary insulinomas were most frequently located within one of the pancreatic limbs, which is consistent with previous findings.^13^ Accurate localization of insulinoma is clinically important, as it directly informs surgical planning and approach.^5^ While previous studies have reported the left pancreatic limb as more commonly affected than the right limb, the current study identified the right limb as the predominant site.^6^^,^^7^^,^^13^^,^^16^^,^^20^ The proportion of cases with suspected metastatic disease (42.3%) was broadly consistent with previous findings.^6^^,^^7^^,^^13^^,^^14^^,^^16^^,^^20^ Ultrasonography was more commonly used (35.1%) in the current primary care study compared to earlier referral care studies, likely reflecting its accessibility and routine use in primary care practice. In contrast, CT, although superior in sensitivity for detecting and localizing insulinoma and identifying metastatic disease, was used less frequently, possible due to limited availability or cost constraints in primary care.^5^^,^^7–9^ The relatively low overall imaging rate, and the infrequent use of CT in particular, might have contributed to underreporting of primary insulinoma localization and an underestimation of metastatic disease. These findings underscore the value of incorporating advanced imaging, preferably CT, into the diagnostic evaluation of dogs with suspected insulinoma in primary care settings.

In our study, medical treatment was used as the sole treatment modality in 65.3% of all cases, which is higher than previously reported in referral populations (33.6%).^4^ The higher rate of surgical intervention in referral settings likely reflects that referred cases, as identified in this study, had increased odds for undergoing surgery compared to receiving medical treatment. This disparity might also indicate that primary care veterinarians are generally more confident in providing medical management than performing complex pancreatic surgery, which remains a relatively uncommon and technically challenging procedure.

Almost all medical treatment in the current study (99.1%) included glucocorticoid therapy, which is consistent with findings of other studies.^5^^,^^6^ Despite diazoxide being the preferred drug to treat insulinoma-induced hypoglycemia, diazoxide was used in only 7.1% of the medically treated dogs in the current study, although this low usage aligns with previously reported percentages that range from 6.7%-14.7%.^5^^,^^10^^,^^14^^,^^21^ The limited use of diazoxide could have been linked to difficulties sourcing diazoxide in the UK and by the high financial cost of long-term diazoxide treatment.^5^ Interestingly, somatostatin analogues were used in 1.4% of the medically treated dogs, of which one case started solely with this therapy, despite several studies reporting conflicting results of using somatostatin analogues as medical treatment for insulinoma.^23^^,^^24^ In our study, 8.5% of the medically treated dogs received toceranib phosphate. Currently, limited studies are available that suggest that toceranib might be effective for medical treatment of insulinoma.^12^^,^^21^^,^^25^ These studies, however, are retrospective and include small heterogeneous groups. Hence, a multi-institutional prospective study is required to investigate the effectiveness of toceranib phosphate in the medical treatment of insulinoma.

In the current study, 48.2% of insulinoma cases received some form of referral care. Most of these cases (67.2%) were referred for both diagnostics and treatment. There are no previous primary care studies that have reported on referral rates for insulinoma cases, but the high rate observed in our study suggests that insulinoma might be considered as a complex condition that poses diagnostic and therapeutic challenges within primary care settings. Insulinoma has a low annual prevalence and incidence in the United Kingdom of 4 in 100 000 and 3 in 100 000 dogs, respectively.^2^ Therefore, it is possible that primary care vets are not fully familiar with diagnostic and therapeutic options. Also, given that contrast-enhanced-CT is not widely available in primary care practices, this might be a consideration for referral.^5^

Regarding risk factors for clinical management and referral, our study found that spaniel breed dogs, referred dogs and dogs presenting with epileptiform seizures had increased odds for undergoing surgery, while dogs aged ≥9 years at time of diagnosis had lower odds. Spaniel breed dogs had higher odds for receiving referral care while dogs aged ≥9 years at time of diagnosis had lower odds. Owners of older dogs might be more hesitant for surgery and referral due to a shorter life expectancy, or anesthetic risks or comorbidities, or a combination of these modalities.^26^ Spaniels breeds like the Cocker Spaniel and English Springer Spaniel are often described as highly interactive, bonded, and behaviorally expressive dogs.^27^^,^^28^ Owners of such breeds might detect subtle behavioral or neurological changes earlier, prompting more rapid veterinary attention and a willingness to pursue referral, advanced diagnostics, and surgery. Additionally, the Cocker Spaniel and English Springer Spaniel are currently classified as Breed Watch category 1 by the UK Royal Kennel Club, meaning that there are no health concerns highlighted for special attention by judges.^29^^,^^30^ Given this general perception of a healthy breed group with good long-term prognoses for many conditions, primary care practitioners and owners might be influenced toward selecting referral and surgery, or surgery, for spaniel breeds, especially because surgery offers the potential for meaningful survival benefit in dogs with insulinoma, as seen in this and previous studies.^10^^,^^13–15^ Epileptiform seizures in dogs are known to have a negative effect on both quality and quantity of life of affected dogs, as well as the quality of life of their owners.^31^ Hence, surgical treatment, offering a rapid restoration of euglycemia and a longer MST, might be preferred in these cases, explaining the increased odds of surgery among dogs with seizures.^5^^,^^6^ Because pancreatic surgery for insulinoma is infrequently encountered in primary care given its low prevalence and incidence, it is likely performed more often in referral settings.^2^ The current study did not, however, assess the associated complication rates of surgeries performed in primary versus referral care practices.

Breed and sex were not significant predictors of survival in this study. In contrast, age at time of diagnosis, treatment modality, and referral were associated with survival outcomes. Dogs aged ≥9 years at time of diagnosis had a higher hazard of death, which likely reflects the natural decline in remaining life expectancy with advancing age, although this association is likely multifactorial.^32^ Dogs aged ≥9 years at time of diagnosis were less likely to undergo surgery or be referred, and both surgical treatment and referral were significantly associated with longer MSTs in this study. Surgically treated dogs had a lower hazard of death, whereas dogs that received no treatment or had an unrecorded treatment, showed a higher hazard of death. Similarly, referred dogs had a lower hazard of death compared to non-referred dogs. The observed associations between treatment modality and survival, as well as between referral and survival, might reflect the close relationship between referral and surgical intervention, supported by our multivariable logistic regression, which identified referral status as a significant predictor of surgery. The finding that surgical treatment confers a survival advantage aligns with previous reports.^4–6^^,^^10^ Additionally, the Kaplan–Meier log rank test also found significant survival differences between treatment categories and referral status categories. Due to the relatively small number of dogs treated exclusively with surgery, survival analyses did not separate cases managed solely by surgery from those receiving combined medical and surgical therapy.

The current study has several limitations that are inherent to research using secondary analysis of veterinary clinical records. The accuracy of diagnoses made in primary care can vary depending on the complexity of the case and the quality of the EHR data. Missing data might have contributed to exclusion of true insulinoma cases or inclusion of cases falsely diagnosed as insulinoma. Further analyses exploring associations between tumor size, metastasis, post-operative glucose levels, survival, treatment, and referral would be valuable, as our study could not investigate this due to the limited available information in the EHRs. The sample size of our study might have restricted detection of subtle associations. In addition, only a proportion of UK primary care practices collaborate with the VetCompass Program so the current results might not generalize fully to all UK primary care practices.^33^

### Conclusions

This study characterized the clinical presentation, diagnostic investigations, use of referral care, survival outcomes, and risk factors associated with treatment decisions and prognosis for dogs diagnosed with insulinoma in primary veterinary care in the UK through the VetCompass Program. The findings of this study underscore that referral and surgical management are associated with improved outcomes for dogs diagnosed with insulinoma in primary care settings.

## Supplementary Material

Kraai_et_al_Fig_S1_Insulinoma_JVIM_aalag045

Supplementary_Table_1_Kraai_et_al_aalag045

## Data Availability

The dataset generated during the current study is available at Figshare: https://dx.doi.org/10.6084/m9.figshare.30294793

## Off-label antimicrobial declaration

Authors declare no off-label use of antimicrobials.

## Institutional animal care and use committee or other approval declaration

Approved by the Royal Veterinary College Social Science Research Ethical Review Board (reference number: SR2018-1652).

## Abbreviations

CI: confidence interval
CT: computed tomography
EHR: electronic health record
HR: hazard ratio
IQR: inter quartile range
MST: median survival time
OR: odds ratio
SD: standard deviation
