# Supplementary figures and images for: Clinical signs, management, and survival of 278 dogs diagnosed with insulinoma under primary veterinary care in the United Kingdom
Source: J Vet Intern Med. 2026 Mar 13;40(2):aalag045. doi: 10.1093/jvimsj/aalag045 (PMC12986752; doi:10.1093/jvimsj/aalag045)

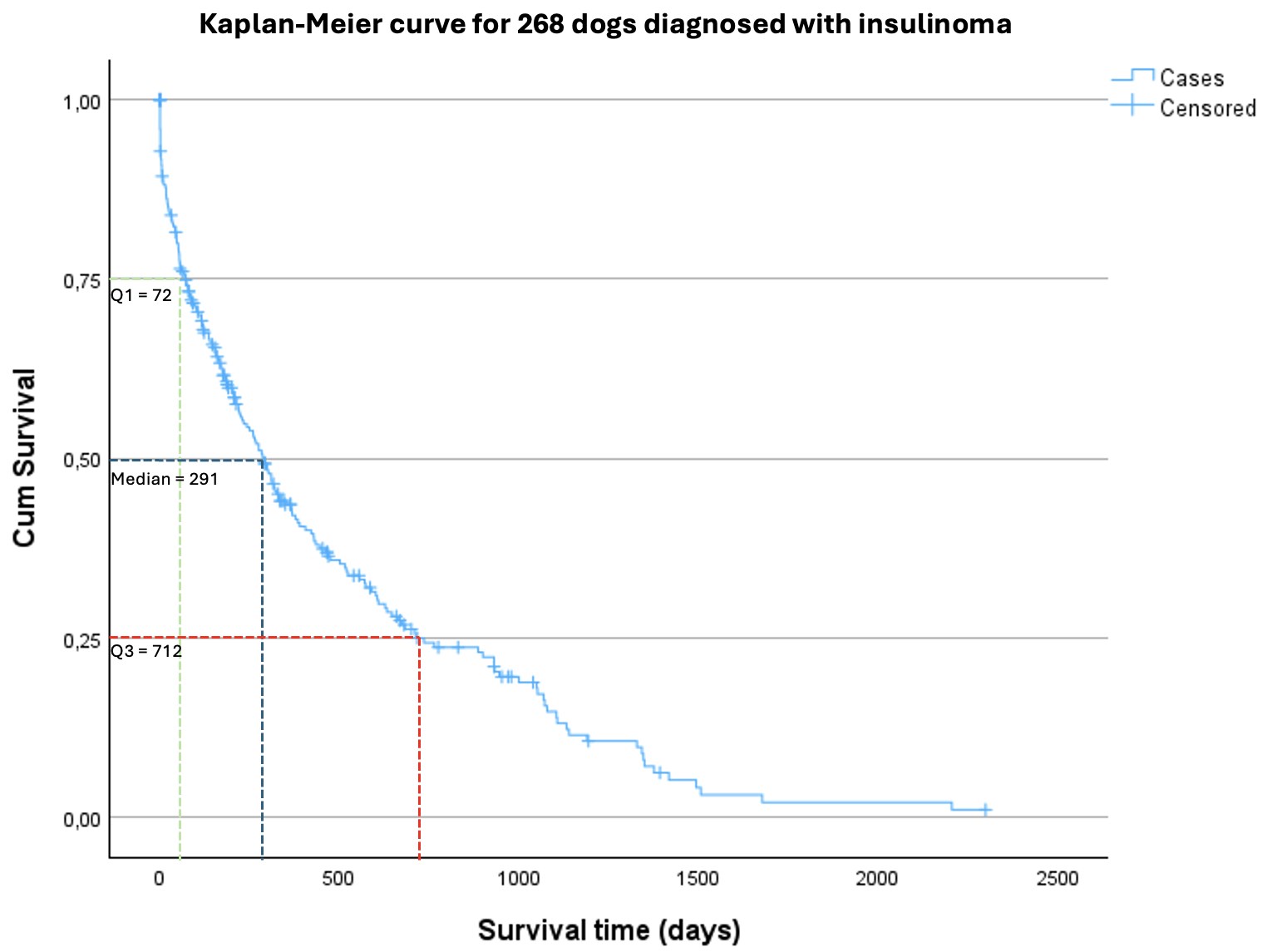

Supplement: Kraai_et_al_Fig_S1_Insulinoma_JVIM_aalag045 [file kraai_et_al_fig_s1_insulinoma_jvim_aalag045.jpeg]
