# Supplementary material for: Clinical signs, management, and survival of 278 dogs diagnosed with insulinoma under primary veterinary care in the United Kingdom
Source: J Vet Intern Med. 2026 Mar 13;40(2):aalag045. doi: 10.1093/jvimsj/aalag045 (PMC12986752; doi:10.1093/jvimsj/aalag045)
Supplement: Supplementary_Table_1_Kraai_et_al_aalag045 [file supplementary_table_1_kraai_et_al_aalag045.docx]

**Supplementary Table 1.** Combination of the four most common clinical signs at the time of diagnosis in dogs diagnosed with insulinoma in VetCompass primary care practices in the UK presenting with a minimum of two out of the four most common clinical signs (*n* = 108).

| **Clinical signs** | **Number of dogs** |
| --- | --- |
| Weakness + collapse/syncope | 21 |
| Weakness + muscle fasciculations | 18 |
| Weakness + epileptiform seizures | 15 |
| Epileptiform seizures + muscle fasciculations | 14 |
| Collapse/syncope + muscle fasciculations | 13 |
| Weakness + collapse/syncope + muscle fasciculations | 9 |
| Epileptiform seizures + collapse/syncope | 6 |
| Weakness + epileptiform seizures + collapse | 5 |
| Weakness + epileptiform seizures + muscle fasciculations | 4 |
| Epileptiform seizures + collapse/syncope + muscle fasciculations | 3 |
